# Testicular cancer from diagnosis to epigenetic factors

**DOI:** 10.18632/oncotarget.20992

**Published:** 2017-09-18

**Authors:** Mariarosaria Boccellino, Daniela Vanacore, Silvia Zappavigna, Carla Cavaliere, Sabrina Rossetti, Carmine D’Aniello, Paolo Chieffi, Evzen Amler, Carlo Buonerba, Giuseppe Di Lorenzo, Rossella Di Franco, Alessandro Izzo, Raffaele Piscitelli, Gelsomina Iovane, Paolo Muto, Gerardo Botti, Sisto Perdonà, Michele Caraglia, Gaetano Facchini

**Affiliations:** ^1^ Department of Biochemistry, Biophysics and General Pathology, University of Campania “L. Vanvitelli” Naples, Naples, Italy; ^2^ Medical Oncology Unit, ASL NA 3 SUD, Ospedali Riuniti Area Nolana, Nola, Italy; ^3^ Progetto ONCONET 2.0, Linea progettuale 14 per l’implementazione della prevenzione e diagnosi precoce del tumore alla prostata e testicolo, Regione Campania, Italy; ^4^ Division of Medical Oncology, Department of Uro-Gynaecological Oncology, Istituto Nazionale Tumori ‘Fondazione G. Pascale’-IRCCS, Naples, Italy; ^5^ Division of Medical Oncology, A.O.R.N. dei COLLI “Ospedali Monaldi-Cotugno-CTO”, Napoli, Italy; ^6^ Department of Psychology, University of Campania “L. Vanvitelli” Naples, Naples, Italy; ^7^ 2nd Faculty of Medicine, Charles University, V Uvalu 84, Prague 5, Czech Republic; ^8^ Department of Clinical Medicine and Surgery, University Federico II of Naples, Naples, Italy; ^9^ Radiation Oncology, Istituto Nazionale per lo Studio e la Cura dei Tumori ‘Fondazione Giovanni Pascale’–IRCCS, Napoli, Italy; ^10^ Division of Urology, Department of Uro-Gynaecological Oncology, Istituto Nazionale Tumori ‘Fondazione G. Pascale’-IRCCS, Naples, Italy; ^11^ Pathology Unit, Istituto Nazionale Tumori “Fondazione G. Pascale”- IRCCS, Naples, Italy; ^12^ Scientific Management, Istituto Nazionale Tumori ‘Fondazione G. Pascale’-IRCCS, Naples, Italy; ^13^ Faculty of Biomedical Engineering, UCEEB, CVUT, Zikova 4, Prague 6, Student Science, H.Podluzi, Prague, Czech Republic

**Keywords:** testicular cancer, germ cell neoplasia, seminoma, biomarkers, epigenetic factors

## Abstract

Testicular cancer (TC) is one of the most common neoplasms that occurs in male and includes germ cell tumors (GCT), sex cord-gonadal stromal tumors and secondary testicular tumors. Diagnosis of TC involves the evaluation of serum tumor markers alpha-fetoprotein, human chorionic gonadotropin and lactate dehydrogenase, but clinically several types of immunohistochemical markers are more useful and more sensitive in GCT, but not in teratoma. These new biomarkers are genes expressed in primordial germ cells/gonocytes and embryonic pluripotency-related cells but not in normal adult germ cells and they include PLAP, OCT3/4 (POU5F1), NANOG, SOX2, REX1, AP-2γ (TFAP2C) and LIN28. Gene expression in GCT is regulated, at least in part, by DNA and histone modifications, and the epigenetic profile of these tumours is characterised by genome-wide demethylation. There are different epigenetic modifications in TG-subtypes that reflect the normal developmental switch in primordial germ cells from an under- to normally methylated genome. The main purpose of this review is to illustrate the findings of recent investigations in the classification of male genital organs, the discoveries in the use of prognostic and diagnostic markers and the epigenetic aberrations mainly affecting the patterns of DNA methylation/histone modifications of genes (especially tumor suppressors) and microRNAs (miRNAs).

## INTRODUCTION

Testicular cancer (TC) is the most common neoplasia that occurs in males between 20–40 years old and it accounts for approximately 1–1.5% of all cancers in men [1–2]. TC develops in testicles and includes several types of cancer, such as germ cell tumors (GCT), sex cord-gonadal stromal tumors and secondary testicular tumors. The incidence rate and mortality change considerably in different geographical areas: the rates are highest in Northern and Western Europe, Northern America and Australia, while lowest rates have been found in South Europe, Central America and, at last, in Asia and Africa [3]. Over the last decades, the incidence of TC in western countries has been increasing, maybe because of an increased exposure to etiologic factors [4]. Genetic and environmental factors play an important role in the genesis and development of TC; in fact, several genes are implicated in its pathogenesis [5] and different environmental factors have been investigated. In the environmental agents there are pesticides and non-steroidal estrogens, such as diethylstilbestrol (DES) [6–7]. There is an association between increased TC risk and maternal smoking during pregnancy, adult height, body mass index, diet rich in cheese are other factors correlated to TC development [8–10]. However, the biological mechanisms involved in TC development are poorly known. Among the risk factors correlated to the onset of disease we can remember: age, cryptorchidism, family history of TC, Klinefelter’s syndrome, personal history of TC, congenital abnormalities and infertility [11–12]. Age represents one of the most frequent factors of TC occurrence; in fact, the highest incidence of GCT has been found in men between 15 and 35 years old [13]. Cryptorchidism is the major risk factor associated with GCT: it deals with the undescended testicle into the scrotum, which remains in the abdomen or groin, and the risk of developing the disease does not change even after surgery to move the testicle into the scrotum [14]. Family history of TC is another factor correlated to an increased TC risk: it has been discovered that the TC risk is significant in men whose father or brother has had the disease. Klinefelter’s syndrome, caused by a chromosomal abnormality, has been associated with TC and many other cancer types [15]. Moreover, a personal history of TC could promote the development of a second cancer. Congenital abnormalities of the testicles, penis or kidneys may contribute to increase the risk of TC [16]. Finally, it has been shown that infertility is strongly associated with TC [17]. The diagnosis of TC can be generally performed both by testicular ultrasound, a very sensitive test to confirm clinical suspicion and in determining where the mass is located (intra- or extra-testicle), and by evaluation of tumor markers. In recent years, many advances have been obtained in TC treatment. Surgical methods have been developed during the years and they represent the best treatment options to treat different types of TC varying depending upon the type and stage of cancer after diagnosis. Treatment options for TC include surgery, radiation therapy, chemotherapy and stem cell transplant. Sometimes more than one type of treatment might be used including chemotherapy and/or radiotherapy [18].

### Classification scheme for tumors of male genital organs

Most testicular tumors (about 90–95%) arise from germ cells to generate the “GCT”, followed by gonadal stromal tumor (5–10%), mixed GCT and secondary tumors. The World Health Organization (WHO) recapitulates the classical histological entities of TCs in seminoma (SE) and nonseminoma (NS) hystotypes. SEs can be detected into three variants: classic, anaplastic and spermatocytic, whereas NSs include choriocarcinoma, embryonal carcinoma, teratoma and yolk sac tumors (YST). Testicular GCT may arise from a non-invasive form of disease named carcinoma *in situ* (CIS): under the microscope these cells appear abnormal although they have not yet spread outside the walls of the seminiferous tubules. CIS does not always degenerate in invasive cancer but it is very difficult to discover it because it often does not involve organ structures; a good way to diagnose CIS is to do a biopsy [19–20]. When CIS becomes invasive, cancer cells spread either to the lymph nodes through either lymphatic or blood circulation. Gonadal stromal tumors can also develop in supportive and hormone-producing tissues, or stroma, of the testicles. They constitute less than 5% of adult testicular tumors and include two main types: Leydig cell tumors and Sertoli cell tumors. These tumors are usually benign and surgical approach is required for treatment, but they become resistant to conventional therapy when metastasize to other organs [21–22]. Mixed GCTs include both SE and NS cells; they grow and spread like NSs, so treatment as NSs is expected. Finally, secondary tumors are not really testicular tumors, but they arise in other organs and then spread to testicle: the most common of them is lymphoma [23]. According to its evolution, three stages of the disease can be distinguished: stage I (the tumor is circumscribed by the testicle), stage II (the tumor has spread to the lymph nodes of the abdomen) and stage III (the tumor has spread to the lymph nodes also with distant metastases in organs such as lung and liver). The initial symptoms are generally mild: in most cases TC appears with a hard mass (sore or indolent) in the scrotum. Sometimes it can happen a diagnostic delay of 2–3 months due to patient reluctance to be examined by doctor or an early diagnosis of inflammatory disease (epididymitis, orchi-epididymitis) and subsequent antibiotic therapy. In advanced disease lumbar pain usually caused by compression of abdominal structures by increased volume of lymph nodes, respiratory disorders related to the presence of lung metastases or lymph nodes of increased volume may be present.

Compared to the latest classification of urinary tract and male genital organs by the World Health Organization (WHO), updates have been done in 2016 that reflect the different behaviour, pathogenesis and tumour biology of similar histological patterns occurring in different contexts. In particular, germ cell neoplasia *in situ* (GCNIS) has been used as a new name for the precursor lesion [24]. This lesion is composed of atypical cells similar to those of SE with enlarged hyperchromatic nuclei, clumped chromatin and prominent nucleoli, aligned along the basement membrane of seminiferous tubules in the spermatogonial niche [25]. Another main change of the WHO classification is the partition of testicular GCTs into two groups: tumours predominantly (but not exclusively) occurring in prepubertal patients, considered not to be derived from GCNIS; and tumours derived from GCNIS, postpubertal-type tumours [26] (Table 1).

**Table 1:** Updates of Germ cell tumour classification in world health organization (WHO)

GERM CELL TUMORS	
GCNIS-derived	Not GCNIS-derived
Seminoma	Spermatocytic Tumor
Yolk Sac Tumor	YST Prepubertal Type
Trophoblastic	Teratoma Prepubertal Type
Embryonal Carcinoma	
Teratoma Postpubertal Type	

In this classification spermatocytic tumour is adopted as a replacement for spermatocytic SE, to avoid confusion with the unrelated usual SE. Trophoblastic tumours include epithelioid and placental trophoblastic tumours analogous to those of the gynaecological tract. The GCNIS-derived tumors display an invasive phenotype where the generation and expansion of tumor cells are limited to within the seminiferous tubules [27–29]. Not GCNIS-derived tumours contain stem cells and cells with various degrees of differentiation toward somatic lineages, thus giving rise to a pleiotropic morphological aspect [30]. In contrast, SEs have a rather uniform aspect, at least at the histological level; therefore, SEs are particularly suitable for investigations of tumor-associated alterations in gene expression.

### Prognostic and diagnostic markers

There are several types of markers for testicular GCTs: serum tumor markers and Immunohistochemical markers. Clinically useful immunohistochemical markers for GCT are genes expressed in primordial germ cells (PGCs)/gonocytes and embryonic pluripotency-related cells but not in normal adult germ cells. They are PLAP, OCT3/4 (POU5F1), NANOG, SOX2, REX1, AP-2γ (TFAP2C) and LIN28. One of the key regulators of pluripotency, OCT3/4, is positive in all CIS, SE, and embryonal carcinoma. Furthermore, it is also expressed in normal adult stem cells and non-germ cell-derived cancers [31]. NANOG is specifically expressed in CIS, embryonal carcinoma, and SEs, but not in teratoma and YST revealing a molecular link between GCTs and the relative embryonic cells [32]. Moreover, HMGA1 and HMGA2 represent valuable diagnostic markers as they are differently expressed depending upon the states of differentiation of TGCTs, with overexpression of HMGA1 in SEs, overexpression of both proteins in pluri-potential embryonal carcinoma cells, loss of expression of HMGA1 in YSTs and of both proteins in mature adult tissue teratoma areas [33–37]. A nuclear transcriptional repressor PATZ1 suggests an impaired function when it is delocalized in the cytoplasm. In human SEs it has been shown that both PATZ1 and HMGA1 cytoplasmic delocalization associates with estrogen receptor β (ERb) down-regulation in human SEs [38–39]. In addition, PATZ1 interacting protein RNF4 is expressed in spermatocytes but not in all tumors including SEs, the highly malignant embryonal carcinomas, YST, and mixed GCTs suggesting that the lack of RNF4 expression could play a role in the progression of testicular tumors [40–41]. Aurora-B expression is another marker used to discriminate the different tumor histotypes; it was detected in all CIS, SEs and embryonal carcinomas but not in teratomas and YSTs [42–44]. Regarding serum markers, those currently used for prognosis are serum lactate dehydrogenase (S-LD), S-alpha fetoprotein (S-AFP), and S-h-chorionic gonadotropin (S-hCG) [45]. LD-1 is related to the characteristic chromosomal abnormality in TGCT, AFP is the main tumor marker used to monitor TC whereas S-hCG is essential in diagnosis and follow-up of hCG producing TGCTs [46]. AFP and β-hCG are expressed not only in germ cell tumors, but also in other neoplasms; specifically, increased levels of β-hCG have been found in neuroendocrine tumors, cancers of kidney, lung, head and neck, bladder and GI tract [47–49], while increased levels of AFP have been found in liver diseases [50–51]; on the other hand, LDH is nonspecific and may be discovered in different conditions both benign and malignant (see Table 2).

**Table 2:** Immunohistochemical markers in TC subtypes

	OCT3/4	HMGA1	HMGA2	PATZ1	RNF4	AUR. B	NANOG	LIN28
**Seminoma**	+	+	–	+©	–	+	+	+
**Embr. carc.**	+	+	+	+©	–	+	+	+
**Teratoma**	–	–	–	+©	–	–	–	–
**Yolk sac**	–	–	+	+©	–	–	–	+
**Choriocarcinoma**	**–**	**n.o.**	**n.o.**	**–**	**n.o.**	**–**	**+/–**	**+/–**

Notes: +, expressed; +© cytoplasmic localization; –, not expressed; +/– variable expression; n.o., not observed.

### Epigenetic factors

During recent years the epigenetic factors have been found to be extremely important in the development of cancer. Indeed, nuclear morphologies are often pleiotropic across a single tumor, reflecting the heterogeneous nature of cancer. These modifications and changes of nuclear structure are key features distinguishing cancer cells from their normal counterparts. Altered nuclear morphology also reflects broad changes in genome positioning and epigenetic changes, which occur during transformation. In particular, in cancer the tight regulation of DNA methylation and the distribution of methyl-cytosine change. Commonly, the heavy methylation in the bulk chromatin is reduced, while the normally unmethylated CpG islands become hypermethylated [52–53]. In testicular GCTs it is important to evaluate DNA methylation in the context of the PGCs from which the tumors arise because PGCs are at a developmental stage where their genomes are highly under-methylated [54–56]. Several studies have demonstrated minimal or no methylation in SEs, and hypermethylation in specific gene promoters of NSs, especially in highly differentiated NSs, suggesting that the degree of cell differentiation may be related to the genome methylation status [57–58]. This phenomenon, in cancer biology, regards GCTs because they arise from cells that, during developmental pathway, change their genome from an under-methylated status to a normally methylated one. Smiraglia et al. show a comprehensive analysis by restriction landmark genomic scanning (RLGS) of the methylation state of an average of over 1,400 RLGS fragments representing CpG islands in both SEs and NSs. They found that the two subtypes of tumors exhibit epigenetic differences; in fact, SEs have extremely low levels of CpG island hypermethylation while NSs exhibit CpG island methylation at a level similar to other cancers of somatic cell origin. In addition, they demonstrate that SEs are more highly hypomethylated in their bulk chromatin than NSs. These epigenetic differences may reflect the normal developmental switch in PGCs from an under- to normally methylated genome. They explain the development of the differential epigenetic phenotypes based on the timing of the transformation events; in details, they believe that the transformation occurs prior to the switch in the methylation state of the PGC genome that creates a CIS with low level of the immunohistochemical marker TRA-1–60 expression and high copy number of chromosomes 12 and 15. This CIS-SE would eventually develop into a SE showing a highly hypomethylated genome with no aberrant CpG island hypermethylation [59]. Mammalian Bre1 complexes (BRE1A/B and RNF20/40) in humans are important for maintenance of genomic integrity, which serves as a barrier against cancer formation. Bre1 factors function as ubiquitin ligases in mono-ubiquitination of histone H2B. This ubiquitination facilitates methylation of histone H3 at K4 and K79; therefore, Bre1 and its homologs have a role in transcriptional regulation. In addition Bre1 acts as a tumor suppressor, augmenting expression of selected tumor suppressor genes and suppressing selected oncogenes. Chernikova SB et al. have demonstrated that Bre1 (human BRE1A/B, RNF20/40) acts as an important suppressor of chromosomal instability (CIN). Moreover, they analyzed BRE1A (RNF20) and BRE1B (RNF40) expression data available from public databases and found significantly lower levels of BRE1A mRNA in human SEs compared with normal testicular tissues. Therefore, Bre1 reduction levels could compromise the genome stability [60]. CIS cells as PGCs and gonocytes, express transcription factors associated with embryonic stem cell pluripotency, such as POU5F1/OCT-3/4, NANOG, T1A-2, MYCL1, GDF3, LIN28-A, DPPA4, DPPA5, KIT and AP-2g. Genome-wide gene expression profiling revealed specific embryonic stem cell-like features of testicular CIS [61–62]. The epigenetic pattern of CIS is associated with an open and permissive chromatin structure based on expression pattern of genes and transcription factors. Histone modifications H3K9me2 and H3K27me3, associated with a restrictive chromatin structure, were absent in CIS cells but H3K4me1, whilst H3K4me2/3, H3K9ac and the histone variant H2A.Z, associated with active and permissive chromatin structure, were abundantly present [63]. This epigenetic pattern indicates that the CIS cells are arrested in the transition between H3K9me2 and establishment of H3K27me3.

In last decades, a new class of small regulatory RNA has been identified: microRNAs (miRNAs). MiRNAs are small non coding RNA (about 18–25 nucleotides) that bind to complementary sequences in the 3′UTR or other regions of their mRNA targets and are able to regulate gene expression at either transcriptional level through induction of mRNA degradation (only in lower vertebrates and plants) or at post-transcriptional level by inhibition of protein translation [64]. In mammals, the complementarity between miRNAs and mRNA target is not perfect: this makes really difficult to identify downstream targets of specific miRNAs; to this purpose, specific algorithms have been developed in order to predict putative miRNA targets. The miRNAs expression profile could provide information on cancer prognosis. Several studies have shown that miRNAs are aberrantly expressed in human tumors and have been identified in different types of biological fluids (urine, saliva, cerebrospinal fluid); therefore, they are considered useful biomarkers in cancer. Voorhoeve et al. carried out studies on the expression of miR-371-3 cluster in testicular GCTs and they found that miR-372 and miR-373 were overexpressed: in particular they showed that miR-372 and miR-373, together with RAS, induced neoplastic transformation in presence of wild-type p53 [65]. p53 protein is a transcription factor that responds to DNA damage by inducing apoptosis and cell cycle arrest [66–67]. It is often inactivated in several types of tumors [68–69]; on the other hand, in testicular GCTs wild type p53 protein was overexpressed but no p53 mutations were detected [70–75]. Moreover, it was demonstrated that p21, a p53 target gene involved in cell cycle block, is not overexpressed in these tumors, which may elucidate the presence of wild-type p53 in this type of cancer [76]. Beyond their role in carcinogenesis of testicular GCTs, miRNAs are involved in the development of PGCs and in spermatogenesis [77]. McIver et al. carried out studies on mice; in details, they showed how miRNAs expression profile changes in different stages of development, especially in the transition between postnatal gonocytes and spermatogonia, which represents a key step in the development of TC [78]. This study led to detect seven differentially expressed miRNAs: miR-136, miR-743a and miR-463* that were over-expressed, while miR-290-5p, miR-291a-5p, miR-293 and miR-294* were down-expressed [79]. The analysis of their putative targets revealed that these miRNAs are implicated in PTEN and Wnt/β catenin signaling pathways and also in CXCR4 pathway [77]. PTEN is a tumour suppressor that exerts its action through inhibition of PI3K signaling, thus its repression leads to increased cell proliferation and potential neoplastic transformation. Moreover, loss of tumour suppressor gene PTEN marks the transition from intratubular germ cell neoplasias (ITGCN) to invasive GCTs. Therefore, its depletion acts as a progression factor in TC having been involved in the inhibition of tumour cell invasive properties [80]. Loss of tumour suppressor gene PTEN marks the transition from intratubular germ cell neoplasias (ITGCN) to invasive germ cell tumors. Both PTEN and Wnt/β catenin pathways are able to regulate the Cyclin D1 action, which was found over-expressed in testicular GCTs and correlates with chemotherapy resistance in these types of tumors; therefore, Cyclin D1 could be considered as a potential target [81]. Afterwards, three of the most expressed miRNAs (miR-291a-5p, miR-293 and miR-743) were chosen in order to detect their targets using a knockdown assay, an alternative approach, through which it was possible to identify IGFBP7 (Insulin-like growth factor binding protein 7), an hopeful gene that can act as positive or negative regulator on cell proliferation and migration according to the environment in which it is found [82–84]. Orum and Lund conceived an innovative method in order to identify miRNA targets implicated in proliferation and germ cell migration and acting on pathways regulated by Platelet Derived Growth Factor (PDGF) and RAC signaling: PDGF is involved in gonocyte migration and proliferation [85], while RAC has been established to be really important for the transmigration of spermatogonial stem cells through the blood-testis barrier [86].

Gillis et al. carried out a quantitative screening of miRNAs on different histological types of human GCTs and they discovered that miR-19a, miR-29a and miR-155 were over-expressed, while miR-133a, miR-145 and miR-146 were down-expressed in testicular GCTs [87]. Syring et al. demonstrated that serum miRNAs can be used as novel and more sensitive and specific biomarkers in TC; in fact, they detected high levels of miR-371a-3p, miR-372-3p, miR-373-3p and miR 367-3p in patients with locally advanced disease [88]. It has been hypothesized that higher sensitivity and specificity of these serum miRNAs could help to identify testicular GCTs. Novotny et al. performed a specific statistical analysis to understand how CIS cells progress and degenerate in malignant tumor by determining whether any changes in gene expression are statistically significant and they discovered that miR-9 and miR-105 levels were high in SE, miR-182, miR-183 and miR-96 levels were increased in SE and embryonal carcinoma, while miR-515-526 cluster was really increased both in CIS, SE and embryonal carcinoma [89] (see Table 3).

**Table 3:** Epigenetic factors involved in TC

EPIGENETIC FACTORS	
DNA METHYLATION	References
•**Primordial Germ Cells (PGCs)** -Seminomas (low levels of CpG Islands hypermethilation) -Nonseminomas (normal levels of CpG Islands methilation) •**Bre1 complexes (BRE1A/B (RNF20/40)** -methylation of histone H3 at K4 and K79	[54–56] [60]
**TRANSCRIPTION FACTORS associated with ESC** (POU5F1/OCT-3/4, NANOG, T1A-2, MYCL1, GDF3, LIN28-A, DPPA4, DPPA5, KIT, AP-2g)	[61–62]
**HYSTONE MODIFICATIONS** - H3K9me2, H3K27me3 (absent in CIS) - H3K4me1, H3K4me2/3, H3K9ac, H2A.Z (present in CIS)	[63]
**MIRNAs** •**Upregulated** (miR-371-3 cluster, miR-136, miR-743a, miR-463*, miR-19a, miR-29a, miR-155, miR-371a-3p, miR-372-3p, miR-373-3p, miR-367-3p, miR-9, miR-105, miR-182, miR-183, miR-96, miR-515-526 cluster) •**Downrelated** (miR-290-5p, miR-291a-5p, miR-293, miR-294*, miR-133a, miR-145, miR-146)	[65, 79, 87, 88, 89] [79, 87]

## CONCLUSIONS

Testicular tumours are rare, comprising 2% of all cancers in men; however, post-pubertal TC is the most common malignancy affecting males aged 15–34 years. Its incidence has doubled over the past 40 years and continues to increase, especially in white men, and if diagnosed early the healing rate is almost 99%. Testicular GCTs are divided into two main histological types, SEs, which resemble the undifferentiated germ cells and the NSs, which resemble both embryonic and extra-embryonic tissues for their ability to differentiate down either pathway. SE and NS exhibit similar cytogenetic abnormalities; the nature of the genomic differences that might account for the phenotypic distinctions between SE and NS are not entirely known. The WHO in 2016 has incorporated the most up-to-date information concerning tumours of the testis and paratesticular tissues. In testicular tumours, the majority of the changes occurred in the nomenclature and classification of GCTs; however, several modifications were also made for non-GCTs.

PLAP, OCT3/4 (POU5F1), NANOG, SOX2, REX1, AP-2γ (TFAP2C) and LIN28 are genes expressed in PGCs that represent the clinically most useful immunohistochemical markers for GCT. Diagnosis of GCT involves the measure by immunoassays of a set of biochemical tumour markers including β-HCG, AFP and LDH and LD-1. AFP and β-HCG are secreted by nonseminomatous tumours, YST and syncytiotrophoblast of choriocarcinoma, whereas LDH is also secreted by SE. The presence of just a few giant cells in a SE may be sufficient to detect β-HCG in serum. Unfortunately, only 60% of patients affected by testicular GCTs have increased levels of these markers. In fact, the interpretation of serum levels of these markers in patients with SE, pure EC and teratoma is sometimes difficult, and many cases are marker-negative. In this light, the mechanism of post-genomic gene expression regulation becomes very promising for an alternative diagnosis. In particular, one of these involves small non-coding RNAs, (miRNA), which are among the smallest (∼22 bases long) RNAs, thus much less prone to degradation. MiRNAs inhibit gene expression by direct base-pairing with transcripts, and one miRNA may have many targets. They act inside the cells, but lately it has become clear that miRNAs are secreted in exosome vesicles that can affect post-transcriptional regulation in a remote recipient cell. Therefore, the can become useful as circulating tumour biomarkers.

Genetic studies show that NSs exhibit variable levels of hypermethylation fragment typical of other tumor types, while SEs lack hypermethylation fragment. Furthermore, the epigenetic difference seen at CpG islands has been demonstrated to also involve the bulk chromatin; in details, SEs show almost no CpG island methylation, in contrast to NSs showing CpG island methylation similar to other solid tumors. Considering that both subtypes arise from PGCs, these epigenetic differences reflect the normal developmental switch in PGCs from an under methylated genome to a normally methylated genome. For normal development, it is necessary to regulate the genome methylation and, therefore, the inability of CIS-SE cells to properly regulate the methylation of their genomes precludes the cells’ ability to differentiate. On the contrary, the ability of CIS-NS to methylate their genome allows them to differentiate, although incorrectly, and the aberrant methylation seen in the tumors is a result of the same type of dysregulation of the DNA methylation system. Epigenetics-miRNA axis may potentially be used to screen semen of men at risk for CIS and testicular GCTs. Currently, no studies about miRNAs in semen have been performed; therefore, further insights are required. To date, specific miRNAs have been tested in several studies in infertile population. According to our knowledge, gene expression is active during spermatogenesis and miRNAs are differentially expressed during differentiation process, so it would be interesting to study the miRNAs expression profiles both of normal and infertile men in order to identify those ones that could be potential biomarkers to diagnose and classify male infertility and for diagnosis and follow up of both CIS and different forms of invasive testicular tumors. A new dawn is arising in the scenario of TC diagnostic and prognostic classification based upon the use of epigenetic markers.
